# Supplementary material for: Emergence of sector and spiral patterns from a two-species mutualistic cross-feeding model
Source: PLoS One. 2022 Oct 19;17(10):e0276268. doi: 10.1371/journal.pone.0276268 (PMC9581386; doi:10.1371/journal.pone.0276268)
Supplement: S1 File — (PDF) [file pone.0276268.s001.pdf]

Emergence of sector and spiral patterns from a two-species mutualistic cross-feeding model

Jiaqi Lin[1], Hui Sun[2], JiaJia Dong[3 *].

1 Department of Computer Science, Bucknell University, Lewisburg, Pennsylvania, USA
2 Department of Mathematics, California State University, Long Beach, California, USA
3 Department of Physics & Astronomy, Bucknell University, Lewisburg, Pennsylvania, USA

* jiajia.dong@bucknell.edu

structures of the colony.

Supporting information

S1 Measurement of interface fluctuations. We develop the algorithm in MATLAB code. To characterize the fluctuating boundaries separating the two species, we define the lattice site p_i along the interface as the following: Each lattice site can take on values 0 (empty), or 1 or 2 depending on the occupant cell type. p_i is *on the interface* if it is occupied by a cell and there are only two unique non-zero values among itself and its four nearest-neighbor sites. The collection of points $\{p_i\}$ defines the interface and its length is L.

For each interface, we quantify the fluctuation along the interface as a function of a sliding window $x \in [1, L]$. Given x, We first find the best linear fit $y(x')$ for the segment connecting from p_0 to p_{x-1}. We then compute the mean square displacement y_0^2 between the interface and $y(x')$. This process is repeated by moving the segment along the interface one point at a time. Then the average of y_i^2 gives us the mean square displacement $\overline{y^2}(x)$ for a given window size x.
