# Supplementary material for: Emergence of sector and spiral patterns from a two-species mutualistic cross-feeding model
Source: PLoS One. 2022 Oct 19;17(10):e0276268. doi: 10.1371/journal.pone.0276268 (PMC9581386; doi:10.1371/journal.pone.0276268)
Supplement: S2 File — (PDF) [file pone.0276268.s002.pdf]

Emergence of sector and spiral patterns from a two-species mutualistic cross-feeding model

Jiaqi Lin[1], Hui Sun[2], JiaJia Dong[3] *.

1 Department of Computer Science, Bucknell University, Lewisburg, Pennsylvania, USA
2 Department of Mathematics, California State University, Long Beach, California, USA
3 Department of Physics & Astronomy, Bucknell University, Lewisburg, Pennsylvania, USA

* jiajia.dong@bucknell.edu

structures of the colony.

Supporting information

S2 Colony patterns with asymmetric mutualism. Our work focuses on the mutualistic cross-feeding case where all parameters are the same for both species. However, the spiral patterns are robust even when the parameters are asymmetric. Below we show a few scenarios where the nutrient diffusion rates and the growth rates are different for the two species. Given the large parameter space, we will reserve a comprehensive study on all possible asymmetric configurations in another study.

As one of the nutrient diffusion rate, D_B (molecules excreted by species 1 to be taken up by species 2), is reduced while keeping D_A the same, we see the system continue to emerge into a stable spiral pattern, shown in Fig.1. When $D_B \ll D_A$ with the same excretion rate, the slower-diffusing nutrient leads to local concentration lower than the Monod constant K_B, thus reducing the effective growth rate for species 2 (blue cells). In this case, species 2 lose out in competing for available growth space and is engulfed by the faster growing species 1, as shown in Fig.1(A). The decrease in one of the nutrient diffusion rates also led to the decrease in overall colony size due to the coupling of individual growth rates $\lambda_{1,2}$ and local nutrient concentration $n_{A,B}$.

When the maximal individual cell growth rate $\lambda_{1,2}$ is varied, we again observe a stable spiral pattern when λ_1 and λ_2 are comparable. In Fig.2(A-B), species 2 (blue cells) have a much smaller maximal growth rate. At the nascent stage of the colony, the limited number of empty lattice sites are taken up by species 1 (green cells), resulting in the engulfment pattern. When λ_2 is increased, we see the emergence of a stable spiral pattern again as shown in Fig.2(C-D). It is worth noting that when the two species have different growth rates, the slower growing cells (blue in Fig.2) develop a wider wrapping branch, which is consistent with what we discussed in the main article.

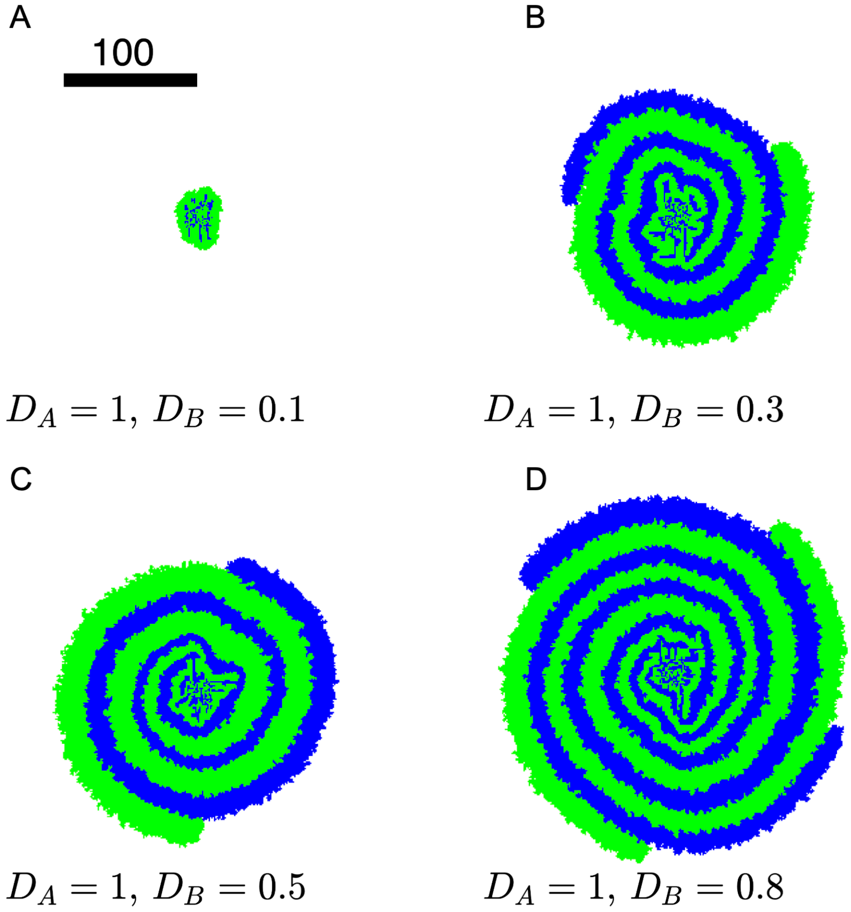

Fig 1. Colony patterns with asymmetric nutrient diffusion rates. Colony snapshots at $t = 1000$. Equal number of species 1 and 2 are seeded in a 20×20 patch with initial density $\rho_0 = 1/4, \lambda_{1,2} = 1$, $\gamma_{A,B} = 1$, $Y_{A,B} = 1$, $D_A = 1$ and $D_B =$ A) 0.1, B) 0.3, C) 0.5, and D) 0.8. Scale bar indicates the width of 100 cells. Green cells are species 1, and blue ones are species 2.

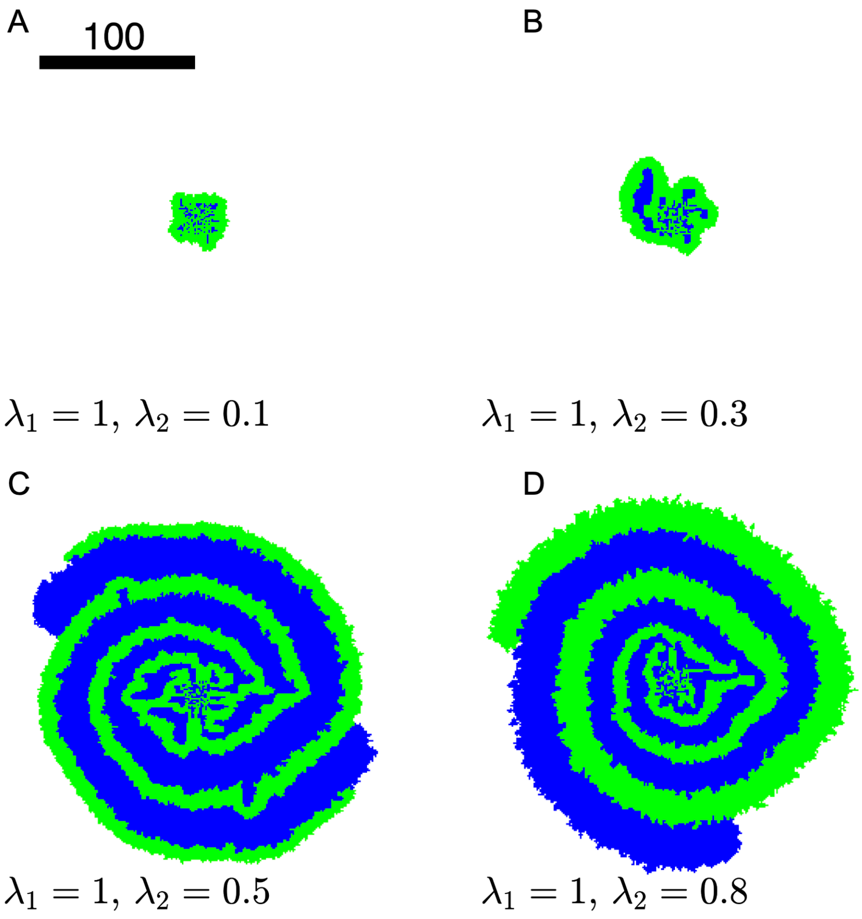

Fig 2. Colony patterns with asymmetric cell growth rates. Colony snapshots at $t = 1000$. Equal number of species 1 and 2 are seeded in a 20×20 patch with initial density $\rho_0 = 1/4$, $D_{A,B} = 1$, $\gamma_{A,B} = 1$, $Y_{A,B} = 1$, $\lambda_A = 1$, and $\lambda_B =$ A) 0.1, B) 0.3, C) 0.5 and D) 0.8. Scale bar indicates the width of 100 cells. Blue cells are species 1, and green ones are species 2.
