# Supplementary material for: Emergence of sector and spiral patterns from a two-species mutualistic cross-feeding model
Source: PLoS One. 2022 Oct 19;17(10):e0276268. doi: 10.1371/journal.pone.0276268 (PMC9581386; doi:10.1371/journal.pone.0276268)
Supplement: S3 File — (PDF) [file pone.0276268.s003.pdf]

Emergence of sector and spiral patterns from a two-species mutualistic cross-feeding model

Jiaqi Lin[1], Hui Sun[2], JiaJia Dong[3 *].

1 Department of Computer Science, Bucknell University, Lewisburg, Pennsylvania, USA
2 Department of Mathematics, California State University, Long Beach, California, USA
3 Department of Physics & Astronomy, Bucknell University, Lewisburg, Pennsylvania, USA

* jiajia.dong@bucknell.edu

structures of the colony.

Supporting information

S3 Link to codes. All codes used to generate the data are published on 365 Github: https://github.com/mr7Jacky/mutualistic-crossfeeding
